# Influences That Retard Evolution in Dental Science

**Published:** 1900-02

**Authors:** S. B. Palmer

**Affiliations:** Syracuse, New York


### INFLUENCES THAT RETARD EVOLUTION IN DENTAL SCIENCE.[1]

BY DR. S. B. PALMER, SYRACUSE, NEW YORK.

Mr. President and Members of the Massachusetts Dental Society,—I thank you for the honor conferred in your kind invitation to read a paper before this society. I cannot express my gratitude for the confidence manifested in leaving the selection of the subject to the writer. This is entitled "Influences that retard Evolution in Dental Science."

It is well at the outset to know why this subject has been considered most important, why it is brought out at the present time, and what is the incentive. The most prominent illustration of retarding evolution in dental science, to my mind, has been the discussion and conclusion of the cause of dental caries. History reports the first paper published on this subject as in 1754–1756. Seven or eight writers are mentioned, which comes down to the time Dr. Miller "took hold of the subject." Since that time Dr. Black and Dr. Williams have united, and apparently have convinced the dental profession that the cause of dental caries is scientifically settled, and any one who ventures to oppose or present other causes are humiliated for presuming to hold out against such talent, learning, and means for demonstration. In financial circles it would be understood as a scientific trust on the etiology of dental caries.

---

[1] Read before the Massachusetts Dental Society, June 7 and 8, 1899.

For this reason the cause of dental caries has been taken up. One advantage of a hopeful understanding lies in the recent progress made in electrical science. Cataphoresis has done much in that line; dynamos, electric lighting, and electrotherapeutics have also contributed to this knowledge. The telephone and wireless telegraphy come in as adjuncts. Thus the present seems more promising than at any previous time.

The incentive is a paper by J. Leon Williams, L.D.S., D.D.S., F.R.M.S., London, England, read before the New York Odontological Society, January 17, 1899. The title of the paper is "Which shall it be, the Empirical or the Scientific Method?" The paper is scholarly, strong in support of science and strong in denouncing clinical experience or science which is based upon empirical practice. There may be grounds for the criticism that it is a plea for a special phase of science, that he is like an attorney retained on a given case to the exclusion of facts upon the opposite side. Let the reader be the judge. In all the papers which have been written upon either side of the question, no other has so greatly helped to establish the so-called "electrochemical theory" upon a scientific basis as the one mentioned. The paper was published in the *Dental Cosmos*, March, 1899. The reason is obvious. Dr. Williams has written in favor of science, he has brought forward many quotations from eminent men, which, from their aptness, as well as from the recognized authority of the writer, fortify his position. I am glad to say that I shall not conflict with any of the findings of Dr. Miller, Dr. Black, or Dr. Williams, nor have I had occasion or disposition to do so. Their work has been done, as all understand, upon the physical plane where experiments can be made, and the results recorded in tables, and done so scientifically that others following the same conditions may obtain like results.

My studies were commenced, as will be seen, previous to Dr. Miller's discovery of microbes. My attention was called to the organic or vital phase of caries, limiting my study to a period of development of the teeth of patients, from twelve to fourteen years of age. My own belief was given that gold—for reasons better known at present—was not the best filling-material to use. Twenty-odd years ago it was unpopular to teach such practice. It was announced in discussion that it "debased gold, exalted amalgam, and degraded the profession." From that time until now some leading scientists have pronounced it a vague idea. I ask all readers to carefully review the paper in the *Dental Cosmos* and see if there appears to be any scientific standing-room for any one at variance with the science therein set forth.

Let us now carefully review Dr. Williams's paper and note the fitness of his quotations, which were made in support of science from a physical stand-point, and see how perfectly they fit organic conditions. They, however, show no understanding of any of the *organic* laws. The first quotation I will make answers two points, first, to ask of my audience the reasonable privileges asked by the author of the paper, and secondly, it seems to show that the three gentlemen agree in their work and findings. "If I may be permitted to speak of my own work as frankly as I would of another's, and without any of that affectation of modesty which is so often used as a thin veneer to cover up the coarsest egotism, I should say that it supplemented and carried to completion in certain directions the work of both Dr. Miller and Dr. Black."

"The first thing which becomes necessary in the effort to induce the dental profession in America to take higher scientific ground is to point out that its present position is not scientific or less so than it should be. In entering upon the subject I quote the definition of science, What is science, and what is it to be scientific in spirit? The simplest and most comprehensive definition of the term is, 'a systematized arrangement of natural facts.' Any man who is engaged in observing the facts of nature and arranging a record of those facts with special reference to their related significance is, just to his scientific labors, a scientific man." Again quoting from the paper, "A fortuitous concourse of facts is not science. Clinical observations do not constitute a science. These things bear the same relation to science that a heap of bricks or roughly quarried stones do to a palace. They are merely the raw materials out of which the building is constructed."

All true scientific work is an effort to learn those constant and persistent conditions which underlie all natural phenomena, and which we call natural laws. Our chief object in acquiring this knowledge is, or should be, its application to life. That life is highest which is lived in most complete harmony with the laws of existence. One might paraphrase an aphorism by Swedenborg, and say "that all real science has relation to life." By this it would seem that science relates to the inner life of teeth as well as to their environments. Another quotation will show the importance of studying nature. "In another department of science we have an eminent example of the rich results which follow the method of accumulating facts direct from nature." It has been stated that Edison has made or has had made and recorded under his instruction more than one hundred thousand original observations from nature. Up to the present time he has probably not been able to make practical use of half of these observations, but he shows that a use for any one of them may be found at any moment. But vast practical adaptations of electricity to human use which he has made, have grown most entirely out of his own first-hand studies of nature. I believe that he has somewhere said that it has cost him almost as much in time to unlearn the errors he had imbibed from books as to accumulate his own original store of facts.

One is reminded by this of that famous saying of Hobbs, "If," he says, "I had read as many books as other men, I should be as ignorant as they are." All great teachers and investigators discourage the use of books as primary sources of knowledge. "Agassiz would have none of them for his pupils." The quotations fit so perfectly the conditions I would have understood, that there is no need of searching for better, if, indeed, such could be found. Hear what Huxley says upon this point: "If the great benefits of scientific training are sought, it is essential that such training should be real; that is, that the mind of the scholar should be brought into direct relation with the facts; that he should not merely be told a thing, but be made to see by the use of his own intellect and ability that the thing is so and not otherwise." In contrasting the book-made man with one who has studied direct from nature, he further says, "Not only is he devoid of all apprehension of scientific conception, not only does he fail to attach any meaning to the words, matter, force, or law in their scientific sense, but worse still, he has no notion of what it is to come into contact with nature or to lay his mind alongside a physical fact and try to conquer it. His whole mind has been given to books and I am hardly exaggerating if I say that they are more real to him than nature. He imagines that all knowledge can be got out of books, and rests upon the authority of some master or other." That expression, "to lay the mind alongside of a physical fact," is to me one of the happiest in the voluminous writings of this great teacher and investigator.

In our review we pass the scoring which was given to college professors and others of high rank and take up "the work of one of our oldest dentists." Though last to be mentioned, one has no occasion to be slighted for non-attention. The writer says, "Of a somewhat different order and demanding more serious consideration are the criticisms passed upon Dr. Black's work in a discussion before the Odontological Society of Pennsylvania, reported in the June number of the INTERNATIONAL DENTAL JOURNAL for the current year (1898). The work of one of our oldest dentists is quoted to say that "the electrical conditions of the teeth are responsible for decay." I was not able to recognize my own language or *ideas* in the quotation from the journal referred to, which reads as follows: "We all recognize that the ideas of the present day have been evolved from the past and, therefore, I thought it might not be amiss to run hastily over the various facts in the experience of the last century and a half regarding the causes of dental caries." Dr. C. N. Peirce commenced with Bourdet and Jourdain in 1754–1756, as before mentioned, and brought the history down to John Tomes. Following John Tomes, "Bridgman, of England, said, 'Decay is due to a chemical electrical action.' He took the ground that the tooth was made subject to the influences of acids by having its electrical conditions disturbed. He was sustained by S. B. Palmer, of Syracuse, who holds the ground to-day that the electrical conditions of the teeth are responsible for decay. He has written several interesting papers in which he says it is impossible to estimate how far this condition changes the character of the dentine, and he maintains that it is an abnormal condition that makes the teeth yield to the influence of the acids of the mouth. This was the condition when Dr. Miller took hold of the subject, etc." The above are the remarks of Dr. Peirce, *his* understanding of the work which had been done by the gentlemen mentioned, given in his own language, not intending to quote the sayings of others, as there is not a quotation mark used in any case. As you read it in the *Dental Cosmos*, one might think the writers had said the things reported, because the quotation marks correctly applied to Dr. Peirce also apply to those of whom he was speaking. The point to be observed is this: Dr. Peirce's remarks were taken as if I had written them. The sentences were analyzed, paraphrased, and logically considered to produce the following: "One can hardly avoid drawing a deep sigh of discouragement in the effort to deal adequately with this sort of thing. It is entitled to a certain amount of respect, and yet it is all so foreign to the scientific spirit and method of working that the situation is much like the old problem put to us in our childhood days, 'If a bushel of wheat sells for a dollar, what will six yards of muslin cost?' There is no relation whatever between one part of his problem and the other part or parts. He first brings in a hypothetical condition as a premise; he then says that it is impossible to tell what the effects of this condition are. If it is impossible to tell anything about it, of what possible significance can it be? It is an abnormal condition, he says, that makes the teeth yield to the influence of the acids of the mouth. This is to me merely a plain statement of the obvious, and there is considerable evidence that several well-known teachers and college professors who took part in this discussion are in agreement with the wisdom of the teaching."

The above quotations from the INTERNATIONAL DENTAL JOURNAL, and their analysis, which was aimed at my teachings, have no more bearing upon this study than the lengthy interpolation of another's views would have to my own. It is upon the supposition that I oppose Dr. Black. The old school-day problem would apply in this case. On turning the leaves we find this sentence: "The opinions expressed were confessedly founded upon clinical experience," although, evidently not upon the facts observed, but upon their interpretation of facts. And have we not all history before us to show that such interpretation may be made to teach just the opposite of what is true when the observed facts are not interpreted by some exact scientific method or principle? May not this account for the interpretation of Dr. Peirce's remarks teaching just the opposite of what he intended? Further on in the paper we read of the vitality of enamel. "I believe that enamel is living," says another college professor, and he continues, "there are some things we cannot measure with a yard-stick; there are certain things we cannot demonstrate with crucible or test-tube." If this means that there may be certain forces and conditions which science has not yet recognized, I should be among the last to deny such a statement. But, then, what bearing does this have upon our manipulation of material things as we understand them to-day? Is the yard-stick to be discredited as an instrument for measuring material things, and shall we abandon the use of crucible and test-tube in our efforts to find out the qualities and relationship of material things? The above is proof that all the investigations are made upon material things that can be scientifically demonstrated. Those who presume to teach that vitality has any part in dental caries are pronounced "fixed in opinions with obvious inward determinations never to yield a point." A careful reader of the paper under review will observe that the principal force of argument of its writer is against the empirical method of teaching which is set in opposition to science.

Speaking of hinderances to scientific progress, we quote as follows: "Another enemy of all true scientific progress is your person of authority. He has acquired notoriety in some special field. He is always to the front when a new generalization based upon new facts in his particular field is brought. He sometimes presents an outward show of deference, but he has an obvious inward determination never to yield a point. Observing him, one is constrained to believe that the force of the most evident facts is lost entirely upon those who are firmly intrenched behind old and fixed habits of thought. Long-continued concentration of mind and purpose in a fixed direction undoubtedly, at least, renders the intellect incapable of perceiving the true relations of things revealed." "The best security for fixedness of opinion is," says Bagehot, "that people should be incapable of comprehending what is to be said on the other side." How does this apply to those who think only on subjects that can be "measured with a yard-stick or demonstrated with crucible and test-tube"? The question is asked, "How is it possible to bear any scientific procedure upon hypothetical conditions which are admittedly beyond the reach of science?" We have never proposed to investigate the unknowable or unthinkable. We are simply trying to establish a solid basis of scientific facts upon which we may work intelligently, instead of empirically, as those must work who "*cannot go over the ground and prove their points, but will have their theories and believe them.*"

"I wonder if any of the gentlemen who are so much given to talking about this mysterious, intangible, vital force have ever carefully examined their own opinions about it?" For one, I will give an answer, yes. I have given years of thought to this subject. I have questioned Nature and received her answers. "The mind has been laid alongside of physical facts and conquered." The reward is that this unknowable, mysterious, intangible, vital force is as well understood as though it had been discovered by experiments in the laboratory. Evolution in science is at a standstill when investigators come to think beyond what may be demonstrated by calcium light or be presented by tables of physical experiments. From the repeated reference to the older men in the profession, and the low scientific rating given them, it would seem that time had been wasted in giving them so much notice. If their teachings are *empirical*, they will soon pass away, young men of science may fill their places, and all will be forgotten.

As we proceed with the review this sentence appears: "Truly it seemed that one must be very alert in these days if one is to contribute anything towards the advancement of knowledge in the dental profession and save one's reputation for accurate statement. At the same time it is noticeable that a large part of the opposition to the new views comes from the older men in our profession. They seem to feel that a change of opinion means the nullification of the work of a lifetime." But I regard that as altogether a wrong position. The condemnation comes not in having taught error, which was really not error so long as nothing better was known, but in clinging to that error after it has been shown to be such. I commend to them this saying of Marcus Aurelius: "If any one can convince me of an error, I shall be very glad to change my opinion, for truth is my business and nobody was ever yet hurt by it. But he that condemns in ignorance and mistake, it is he that receives the mischief." "We would at least wish to see the older men in our profession content to rest with work well done. We would gladly honor them and ask them to accept our full and cordial acknowledgment of the faithful way in which they have marched forward under great difficulties. They have made wonderful progress, although handicapped in many ways and carrying heavy burdens. But must the universe stop with their passing? Must the deluge which ends all, come upon us as they go? Is it not our business also to put the hand of progress forward a little on the dial of time? Why can they not accept the situation gracefully, and frankly admit that they have not exhausted the possibilities of heaven and earth? But whether they will do this or not, Nature smiles upon them serenely, turning the page of her great book to a new problem, calls out the younger class."

As one of the older members referred to, and one who under other circumstances might feel complimented for the generous credit and distinction bestowed above others, he has fixed upon this occasion as the time to file his conclusions in the archives of the Massachusetts Dental Society for the dental professor's perusal, and respect fully dedicates the work to the young men in the profession. It is hardly possible that you will be called upon to carry the burdens of the older men who have watched dental evolution for forty and some for fifty years. As an individual, I have known and been made to feel what it has been, and still is, to stand for my convictions. Early in professional life, when dental literature was rare, and dental societies beyond reach, there was more time for thought and study of nature. The outcome was a theory which was not in harmony with established practice. The theory was given to the profession, and I was advised to *retract* and save my professional reputation, and if I did not, the consequences were defined. Denial of a scientific standing has been firmly adhered to, and you have just heard the latest. Kind offers for giving up, excuses for teaching errors, and finally a challenge which few would care to accept, and quite probably it was expected that no one could be found to accept, and thus would end a life-work in disgrace to empiricism and triumph to physical science. Here are the conditions in the challenge, "We show you that lactic acid is formed by certain micro-organisms in contact with enamel. We show you many appearances of decaying enamel which are identical with appearances produced by artificial application of lactic acid out of the mouth. And yet you go on talking about the absence of a certain vague, vital force, or certain electrical conditions of the mouth as being responsible for dental caries. We ask you to abandon this position, or come forward and astound the scientific world by your announcement of the discovery of some hitherto unknown principles of chemistry."

I wish it understood that in my own mind there has never been any clash between my views and the findings of Dr. Miller, Dr. Black, and Dr. Williams. What has been claimed by them for their investigations has been granted. Exceptions are taken to limiting all knowledge of the causes of dental caries to physics. As the review has shown, my own work which relates to organic matter and vitality has been rejected by science and classed with empiricism. Therefore, until the dental profession fully recognizes this work as scientific, I will gracefully take my place alongside of editors, college professors, and eminent teachers, and wait until a sufficient number of facts have been obtained from clinical practice to be entitled to a standing upon a scientific platform. One cannot but feel responsible, in a measure, for teaching error, and thereby leading others to a point of humiliation. Science is in evolution and knowledge is gained by thought. The limit to knowledge is bounded by capacity of comprehension.

It has been plainly stated in the paper that investigations have been made upon inorganic matter which will admit of physical demonstrations. On the other hand, this study has been limited to teeth in a state of development, in which state the pulp plays an important part. This condition cannot be marked accurately by years. Upon an average, fifteen years would be considered a low estimate for the time children's teeth, or those older which have approximate cavities, could be safely filled with gold without liability of recurrent caries. Close observers will recollect the differences between incipient decay under fillings in children's teeth and those of adults. The former are marked by a brown or yellow shade seen through the enamel, which shows extension over all dentine; while in normal teeth the lines of caries are more clearly defined, darker in color, and not so deep, being the result of imperfect manipulation, while spots are seen through the enamel. I wish it understood that in any case imperfect manipulation introduces the conditions which are so ably supported by science in the paper before us, the truth of which I do not question. Let us consider how caries progress as beneath gold fillings unaided by micro-organisms or lactic acid. From the first office of the pulp of a tooth to the finish of the dentine, the dentine is under vital influence. Nature works upon a principle of consonance of potential, perhaps better understood by polarity, which in a tooth or the whole body would be this: The body is covered by the skin; all within belongs to the negative pole, the positive pole being outside, such as sunlight, warmth, thermal changes, etc. Nature builds up the body from within, using lime, alkali, and such elements as are necessary to complete the teeth. As above mentioned, the functions of the pulp are such as to intelligently direct the construction of the tooth.

In normal dentine all that is necessary to arrest decay is to prepare the cavity and fill with gold when conditions are favorable; and here I will add that gold is the best material in use when adapted to favorable conditions. Now let us consider the unfavorable conditions in the teeth of young people; remember that youth is not limited to a definite age. Teeth that commence to decay on approximate surfaces should not be filled with gold as early as those well enamelled which are attacked with caries arising from fissures or imperfect closing of enamel. Some cases are slower in calcification than others; while a non-conducting filling would allow nature to complete her work, a conductor of thermal changes would check the progress, and the result would be that the portion in contact with the metal would become devitalized beneath the filling, even though the enamel should exclude the lactic acid.

At the opening of the Ninth Medical Congress at Washington, in the address of welcome, the speaker mentioned many modern appliances which aid in medicine and surgery, and added, "with all these give Nature a chance." This remark applies to dentistry to-day.

Obtundents, anæsthetics, cataphoresis, the mallet, are all liable to interfere with nature. Nature rejects mineral food generally by taste, and the above agencies are on the same line and too often forced. Nature tolerates the farmer in tempting the appetite, to fatten his pigs, even the stuffing of turkeys for Thanksgiving; but the dentist often manifests causes for rebellion in stuffing hyperdermically, or the forcing of drugs electrically, as well as malleting gold upon vital dentine. It is all upon a line of shooting civilization into the Filipinos, dangerous to vitality.

In speaking of the influences of gold upon sensitive dentine, thermal change has been used instead of electricity. In the evolution of electricity from mineral to animal bodies we use the same terms, and this introduces the most embarrassing feature of this paper. So far as I know, no one has attempted to formulate or even to state this doctrine of the evolution of forces or laws from the mineral plane to the animal plane, and therefore the embarrassment by being driven to speak of what I have said or done. The discovery, if such it may be called, is a scientific cable-line running from the mineral plane up and into the plane of life, through intellect, reason, etc. I say cable because of the many strands therein.

We will first take up matter, force, and law, known upon the lowest plane. This knowledge is gained in the laboratory, where we have all the material forces and laws as a study. Force is the life of matter; that force is electricity. It is brought to bear upon atoms, and they combine in definite proportions, which is a manifestation of law. The chemist understands that water is $H_2O$ the world over; for his convenience he crowds nature and gives us $H_2O_2$, and at the first opportunity the imprisoned oxygen escapes. Upon this plane, by direction of this life-force, crystals appear, each after its kind. Beauty and variety can be seen in frost-work, even in a snow-flake or in flowers upon the window-pane. Leaving the first appearance of life to the biologist, we mention that vegetable life is supported by mineral food in the absence of that more easily digested, and by the unfolding forces and aid of vegetation, food for animals is prepared. This is a wonderful change; but, being so common, little thought is given to it, and apparently no thought to evolution of matter, force, and laws, to correspond with vegetable matter. Bear in mind there must be a Creator; that knowledge comes to every soul as it can comprehend nature and nature's laws. There is much that is unknowable to the masses which would have been known if man had been taught to think independently rather than to depend upon others to think for him. I do not expect that the radical thoughts here expressed will be received at once. I have given them as they have come to me, "by laying the mind alongside of physical facts and striving to conquer them." No doubt unusual opportunities have been offered to aid in studying nature. They have been improved, and the results are now offered for progress in dental science. One must feel that there has been a mistake in denying this study a scientific investigation, and so much labor bestowed upon the physical aspect. This study has not been wholly confined to dentistry, and the following, which does not, I do not offer as established science. There has been a great desire to establish a scientific connection between atoms and intellect, physics and metaphysics. I do not know that the line has ever been distinctly traced, showing Nature's method of communication with man, showing that man works out what Nature has wrought within. In familiar language, "God created man in his own image."

The next problem is, how does Divinity inspire humanity? Turning to our review we read, "I wonder if any of the gentlemen who are so much given to talking about this mysterious, intangible, vital force have ever examined their own opinions about it? The origin of all this so-called vital force, as Tyndall so ably demonstrated more than twenty years ago, is from that great reservoir of inorganic force, the sun." From the time this announcement came to my notice until now, it has been undergoing evolution, and the facts announced by Tyndall at that time show that he is on the same plane with Dr. Miller, Dr. Black, and Dr. Williams; neither go higher than physics. If Tyndall entertained higher views he wisely kept them to himself. The world was not ready to receive them.

First let us analyze the energy which is given out by a beam or sunlight. Physically speaking, the sun imparts to matter according to its condition to receive all the energy known in physics, of which electricity is the most prominent, and it is the one "live wire" which, like Jacob's ladder, reaches from "earth to heaven." I believe that all revelation from the Creator to man is through electricity in its various phases; commencing with physics and observing its evolution as it appears upon the vegetable and animal planes, we call the change evolution. A more correct view would be to credit evolution with raising matter from mineral to animal bodies, and to say that the sun shines alike upon all conditions of matter, and that matter gives expression to the Divine gift, which is life according to its condition or state to express it. Electricity is the "breath of life" shown in elements, by the order and proportion of crystals, each after its kind; that is, the crystal is the environment of the life which fashioned it. Beauty is also displayed in frost-work.

Vegetable life follows in evolution, minerals are raised up to organic compounds, and in this marvellous change the same laws and forces have undergone evolution to fit the new condition. Life finds material to environ itself. Every seed, bulb, or twig arranges material for a body suited to its needs and purposes, even the same soil or sap being used to accomplish this purpose. A bud grafted into a crab-apple stock would bear a pippin, a sweet or sour apple, according to the life in the bud. Upon the same principle a pulp fashions a tooth in size, form, and color to match a corresponding one. While the pulp derives its life and material from the common stock in the body, it has the designing power in itself. Whatever interferes with its methods is abnormal. At the age of fourteen years teeth that are prone to decay on approximal sides are not sufficiently protected. This is no argument against the lactic acid cause; it is in harmony so far as the physical causes are an influence. But when the vitality and instinct of the pulp are considered new conditions arise. First, nature in the work of organization in vegetable or animal compounds combines elements in various proportions and retains the same until the vital principles are disarranged. To put metal into the dentine of a young tooth, or into dentine which contains organic matter sufficient to render it a conductor of thermal changes, is unnatural. No more lime-salts are deposited at that point so long as the current is reversed or turned back. Gutta-percha, being less conductive, allows calcification to continue and produce a tooth of normal structure. One point is overlooked, and that is heat. In animal bodies normal heat, or the limit of heat, ranges between freezing and 105°. Vegetable life places it between zero and the boiling-point of water, or near this line. It is easy to see that drinks or food when taken into the mouth at a temperature of 150°, in cases of metal fillings, raises the temperature quite above the normal standard. The effects occasionally appear under large gold fillings in bicuspids, which seem perfect and have done good service for several years. If on account of pulp disturbance such fillings are removed, it is found that the lime-salts have been taken back, causing pulp exposure. It has been stated that some conditions arise where the teeth decay less rapidly, where pulp devitalization has been resorted to, the vital action being cut off from the portion within the enamel. Also by slow abrasion teeth may be ground to the gums, the pulp receding and secondary dentine taking its place; that is, where the irritation has not turned back the current of supply. Still other demonstrations are found where clasps have irritated the neck of the tooth and worn the dentine. Nature, to meet the invasion, produces pulp-stone upon the side most worn. It seems that we have facts enough to establish science upon the vital side of dental caries. I wish to claim one point, that vital teeth decay beneath gold fillings, independent of the causes so ably investigated and pronounced settled from the physical stand-point. When the natural organic construction of dentine is bruised by harsh gold or malleting, or where by heat the pulp functions are disturbed, decomposition sets in. This is seen through the enamel where gold has been inserted in young teeth for a year or less. The fact is, that a gold filling which passes through enamel into dentine in any tooth is, while mastication of food is going on, an electrode, becoming charged with electricity and discharged when the dentine is a conductor. In a tooth of normal structure it is not a conductor. Aside from the heat above 105° there is electricity that passes through the gold, which is not tolerated by nature, in contact with dentine. Lime-salts are dissolved by action of the pulp. Before Dr. Miller announced his discovery I had tried the following experiment, which convinced me that the first principles of dental caries were coexistent with *creation*, and was enrolled in the law that acid dissolves lime-salts. The enamel was polished on opposite sides of a molar and the tooth suspended in water, in which some carbonate of soda had been dissolved to preserve alkalinity. The suspension was from a clamp of insulated wire, the points resting upon the enamel and the other ends were put in circuit with two or three cells of battery. In less than twenty-four hours the positive electrode had commenced a cavity in the enamel, while at the negative pole there was a ring of salts but no mark upon the dentine. Another test for acid was as follows: a piece of litmus-paper wet with saliva was laid upon a platinum plate which was in connection with the battery, and an electrode, forming the other pole, was slowly traced over the paper, showing in red lines or characters that oxygen was associated with acid. Bear in mind that decomposition of water is quite different by the vital process from what it is by the physical.

Having spent considerable time upon the vitality of the pulp, let us consider and further pursue our study of evolution and of animal electricity as it is manifested in mind. From my first interest in this study until the present time, there were not facts enough known in vital electricity, all combined, to give an intelligent illustration. First, the mind communicates or corresponds with other minds through several of the senses,—by sight, sound, feeling, etc., —and by writing or printing. In writing, the fingers holding the pen are guided by the mind, the characters are translated by the receiver, and the message becomes knowledge or intelligence to another mind or person. The first electric telegraph worked upon the same principle: the fingers at the key put upon the wire waves of electricity, which were converted into magnetic force, leaving impresses upon a slip of paper. The ear of the operator caught the sound, and paper was discarded, the sounder taking the place of the register. The telephone teaches a more important object-lesson; that is, the voice, the effect of wave-sound, which is thrown into space by vital energy as the wireless telegraph, sends out physical waves. Speaking into the phone causes vibrations in a metal disk which is in consonance with a similar one at the terminus; thus the mind and thought of one person employ the organs of speech, which are vital organs to produce wave-sounds, which are converted into electricity that conveys the intelligence, delivering it to the ear and understanding of one far away. Electricity, as generally understood, is interconvertible into heat, light, chemical affinity, magnetism, etc. It will in time be analyzed, and added to it will be life, intellect, will, thought, and all that belongs to man to know. Man, we are told, has come up through the stone age, has passed the iron age, and now is in the steel age. There is great promise that the near-by century will be an "age of reason," an age when man will better know himself and know more of life, natural laws, and divinity.

It might naturally be asked what this digression has to do with dental science. It relates to the phase of science which treats of life; it is a part of knowledge which is connected with a certain vague, vital force hitherto unknown, that is called for in the challenge. This writing is not a reserve, an unheard of principle brought in to astonish opposition, but principles which have been promulgated for years past. Dentistry has given to medicine and the world some valuable discoveries; it may yet add to physiology some new discoveries, and the time seems ripe for it.

It may be instructive to some present to know how these new principles have been discovered. That is the evolution of natural laws and forces to meet the conditions of life and organized matter.

First, there was a desire for knowledge of electricity and chemistry in boyhood days. As country district schools afforded no opportunity for such study, all that could be done was to read such books as the times offered. In 1847 I commenced to wear a silver plate, —all temporary plates were then made of silver. It was from the chemical action of that plate upon various kinds of food that the thought came to mind that *taste* was from vital or organic electricity. To commence the study, I constructed two galvanometers differing in sensitiveness, to meet demands. About three years were spent in study, mostly in the laboratory, some in the mouth. This was learned, and the facts were noted, that food, such as taste and habit direct to be masticated in pairs, or at the same time, were positive and negative, or were used with fluids which produced the same effect. All food which was browned, roasted, or broiled greatly increased the deflection of the needle when tested in the mouth or in the laboratory, saliva being used as fluid. Acid fruits, vinegar, etc., also increased the current. As the current seemed to be of the same nature in either case, the instruments were of no avail in testing the vital current which was believed to exist.

The first important discovery was that vital electricity, which was revealed in taste, became electricity proper when in touch with metal or carbon conductors, and its vitality vanished. Blood, on leaving the artery, loses its life principle. I can see no way that this discovery could have been made without the wearing of silver. Gold, vulcanite, and aluminum plates were used, each teaching lessons relating to respective conditions, but not in connection with vital electricity. Silver worn in the mouth is an unstable element. Its potential is changed in masticating food with every touch or change of acid or alkali, sulphur, or chloride of sodium. Thus the plate became a vital electro-chemical galvanometer, and probably it was the only one ever consulted in the interest of science. First the line was distinctly drawn between mineral and vegetable compounds as food, or even mixtures of the same. The study became as interesting as that of the microscope, and as scientific; still it is rejected because it cannot be demonstrated before an audience. One point of information gained was the effect produced on food by roasting of coffee, toasting bread, broiling meats, salting almonds, popcorn, etc. The effect, except the salt, was to add carbon in the right quantity, which is on the right side of nature's line between vegetables and minerals as food. Browning does not disorganize the organic structure in any of the articles of food mentioned; burning does. Nature detects the difference by taste. Silver increases the action or taste, sometimes not disagreeably so, but more often otherwise. The sulphur in eggs converted the vital taste into a metallic taste, or bordering upon physical electricity. Carbon from burnt food also increased electrical taste.

Any metal or metallic liquid caused the same sensation that one experiences when placing zinc in the mouth with any other metal. The idea that cider or beer tastes more sparkling when drunk from a pewter mug is not fancy, but scientific. The action of the liquid charges the metal with electricity, and when the beer flows over the edge of the mug it becomes electrified and imparts a thrilling sensation to the organ of taste that could not be experienced in the use of porcelain or glass. This knowledge caused the disuse of the galvanometer to test currents of vitality. It would demonstrate the physical current in an experiment correctly, but could not touch taste. Gold gives but one side of the question of vitality. It being negative and not acted upon, it only acts upon food which is positive in some instances. It detects metals in solution of the liquids in canned goods, but not like silver. Coffee and broiled meats, being also negative, produce very little effect. Rolled or swaged aluminum is by far the most natural for a dental plate. It is not often that any food or material is taken into the mouth that changes taste. Cast aluminum that has been alloyed to aid in casting is not free from objection. It may be interesting to relate my experience in wearing such a plate for one night only; that is, under the same conditions. I volunteered for a clinic at a dental convention, and came out all right, with a cast plate so formed as to cap over a molar on either side to increase the bite and prevent elongation of the teeth. The articulation below was upon a bridge on either side, with gold-crown surfaces. The plate was worn through the day all right, except the electrical taste with which I was familiar. On taking a ride in a street-car in the evening I discovered that all the street lights were demoralized, constantly flashing, but irregularly. It was not long before I learned the cause was not at the central station dynamo, but in an intermittent current in my mouth. By closing the jaws gold and aluminum came in contact, and the flash occurred; while the contact remained closed or when open, all was steady. That was the only instance I have known of vital electricity being converted into light. I do not claim that it was, but somehow the shock acted upon the optic nerve to produce the phenomenon described. The burring out of the metal cap and supplying the cavity with vulcanite corrected the flash light. By opportunities of this kind I became convinced that vital electricity is converted into physical in passing through any metallic or mineral conductor. And still another principle is, all physical currents in use in electro-therapeutics may be regarded as mineral, all regarded for food tolerated to a limited degree and no further.

It is to be regretted that this crisis has been forced upon the profession at this time. To meet the occasion much of a personal nature has been written in explanation that otherwise would not have appeared. The terms offered were to abandon a life-work of thought and study, and thereby acknowledge having taught error; or produce evidence in support of the theory which has been pronounced vague. All differences arise from the conclusions obtained from two distinct studies, one physical the other vital. It is plainly stated in the discussion that life and its relations to matter have not been considered, because it cannot be demonstrated. The work which has occupied my time commences where that which it has supposed to antagonize leaves off. Opportunities, as before stated, have been improved, and the teachings from nature seem as real as though the knowledge had been obtained from physical experiment. To do justice to the discussion quotations were taken from the paper in the *Dental Cosmos*, which are able supporters of science and apply equally well to organic and physical science.

No higher compliment can be paid the writer than to express my feelings towards any and all who have believed they have been working for the advancement of dental science in opposing the electrochemical theory, than by quoting the apt and well-chosen language of the paper reviewed. "I do not wish to see this chosen profession of ours content with a lower level than rightfully belongs to it. I want to see it take its place among the first, and therefore I say come, let us join hands, and let all petty jealousies and small ambitions be shamed into silence in the presence of a noble enthusiasm for real progress, which shall fill each and all of us."

